# FAK promotes stromal PD-L2 expression associated with poor survival in pancreatic cancer

**DOI:** 10.1038/s41416-022-01966-5

**Published:** 2022-09-22

**Authors:** Catherine Davidson, David Taggart, Andrew H. Sims, David W. Lonergan, Marta Canel, Alan Serrels

**Affiliations:** 1 Centre for Inflammation Research, Queen’s Medical Research Institute, Edinburgh BioQuarter, University of Edinburgh, Edinburgh, UK; 2 Edinburgh Cancer Research, Institute of Genetics and Cancer, University of Edinburgh, Edinburgh, UK; 3 Hospital for Small Animals, The Roslin Institute, The Royal (Dick) School of Veterinary Studies, The University of Edinburgh, Roslin, UK

**Keywords:** Cancer microenvironment, Cytokines, Immune evasion

## Abstract

**Background:**

Pancreatic Cancer is one of the most lethal cancers, with less than 8% of patients surviving 5 years following diagnosis. The last 40 years have seen only small incremental improvements in treatment options, highlighting the continued need to better define the cellular and molecular pathways contributing to therapy response and patient prognosis.

**Methods:**

We combined CRISPR, shRNA and flow cytometry with mechanistic experiments using a Kras^G12D^p53^R172H^ mouse model of pancreatic cancer and analysis of publicly available human PDAC transcriptomic datasets.

**Results:**

Here, we identify that expression of the immune checkpoint, Programmed Death Ligand 2 (PD-L2), is associated with poor prognosis, tumour grade, clinical stage and molecular subtype in patients with Pancreatic Ductal Adenocarcinoma (PDAC). We further show that PD-L2 is predominantly expressed in the stroma and, using an orthotopic murine model of PDAC, identify cancer cell-intrinsic Focal Adhesion Kinase (FAK) signalling as a regulator of PD-L2 stromal expression. Mechanistically, we find that FAK regulates interleukin-6, which can act in concert with interleukin-4 secreted by CD4 T-cells to drive elevated expression of PD-L2 on tumour-associated macrophages, dendritic cells and endothelial cells.

**Conclusions:**

These findings identify further complex heterocellular signalling networks contributing to FAK-mediated immune suppression in pancreatic cancer.

## Introduction

Pancreatic cancer is currently the fourth leading cause of cancer death in both the United States [1] and Europe [2], and is expected to become the second most common in the developed world within the next decade [3]. Despite extensive clinical testing of potential cancer therapies, including immunotherapies, major advances in the treatment of patients have not been forthcoming [4, 5]. Therefore, an improved understanding of the key pathways/mechanisms that contribute to poor patient outcomes and resistance to therapy is needed if we are to reverse this trend.

Pancreatic Ductal Adenocarcinoma (PDAC) is the predominant histological subtype of pancreatic cancer, occurring in 90% of cases [6]. Inflammation plays an important role in its development and progression. Both murine and human PDAC tumours are extensively infiltrated with a variety of immune cells that not only contribute to establishing a highly immuno-suppressive tumour microenvironment (TME)[4, 7–12] but also promote PDAC development and progression via hematopoietic-to-epithelial cell signalling [13, 14]. The importance of the TME is further underlined by observations correlating various constituents to patient outcomes. For example, the intratumoural density of myeloid-derived suppressor cells (MDSC), macrophages and regulatory T-cells (Tregs) has been linked to poor survival [9, 10], and the ratio of Th2 (GATA3^+^) to Th1 (Tbet^+^) polarised CD4^+^ T-cells in post-surgical samples has been identified as an independent predictive marker of PDAC patient prognosis [15]. Malignant cells play a central role in orchestrating the composition of the PDAC TME through secreting a range of soluble factors, including chemokines and cytokines, that drive immune cell recruitment and differentiation to promote immune suppression [4, 8, 12, 16–18]. Identifying therapeutically exploitable molecular pathways that regulate paracrine signalling between malignant cells and the TME will therefore likely represent an important component of any therapeutic strategy aimed at unlocking successful immunotherapy in PDAC.

Activating mutations in the KRAS gene, which occur in over 90% of PDAC, drive malignant transformation and tumour-promoting inflammation [19–22]. Previously considered undruggable, inhibitors targeting KRAS are now emerging, with both KRASG12C and Pan-KRAS inhibitors now in clinical development [23]. These inhibitors have shown promising signs of activity in pre-clinical cancer models and early phase clinical trials. However, the development of resistance has been observed in both the pre-clinical and clinical settings, and it is likely that drug combinations will be more effective than monotherapy treatment. Therefore, the identification of downstream effectors or alternative druggable molecular targets that regulate paracrine signalling to control immune suppression remains important. In this context, recent studies have identified the non-receptor protein tyrosine kinase Focal Adhesion Kinase (FAK) as a potentially promising therapeutic target that regulates the fibrotic and immuno-suppressive PDAC TME, rendering genetically engineered and transplantable mouse models of PDAC sensitive to immunotherapies [17, 24]. FAK is hyperactivated in human PDAC [17], and FAK inhibition using either a selective small molecule inhibitor or genetic ablation can impact chemokine and cytokine expression in multiple cell types, including PDAC [17, 25, 26]. As a consequence, FAK inhibitors are now being tested in combination with immune checkpoint blockade in patients with pancreatic cancer in several ongoing clinical trials (clinicaltrials.gov NCT02758587, NCT02546531, NCT03727880).

Here, using a murine model of PDAC, we identify a novel role for cancer cell-intrinsic FAK signalling in regulating the expression of the immune checkpoint ligand Programmed Death Ligand 2 (PD-L2) in the tumour stroma. We show that high PD-L2 expression in human PDAC is a prognostic marker of poor patient outcome and is associated with tumour grade, clinical stage and molecular subtype. Mechanistically, we find that FAK-dependent secretion of interleukin-6 (IL6) from *LSL-Kras*^*G12D/+*^*;LSL-Trp53*^*R172H/+*^*;Pdx-1-Cre* murine pancreatic cancer cells can amplify interleukin-4 (IL4) induced expression of PD-L2, but that IL6 alone cannot promote expression of PD-L2. We further show that CD4^+^ T-cells expressing IL4 are present within the PDAC TME but are not regulated by FAK. These findings identify a novel role for a FAK-IL6 signalling axis in amplifying the expression of pathways associated with immune suppression and poor patient prognosis in PDAC.

## Results

### FAK promotes pancreatic tumour growth associated with increased PD-L2 expression

**Fig. 1:**
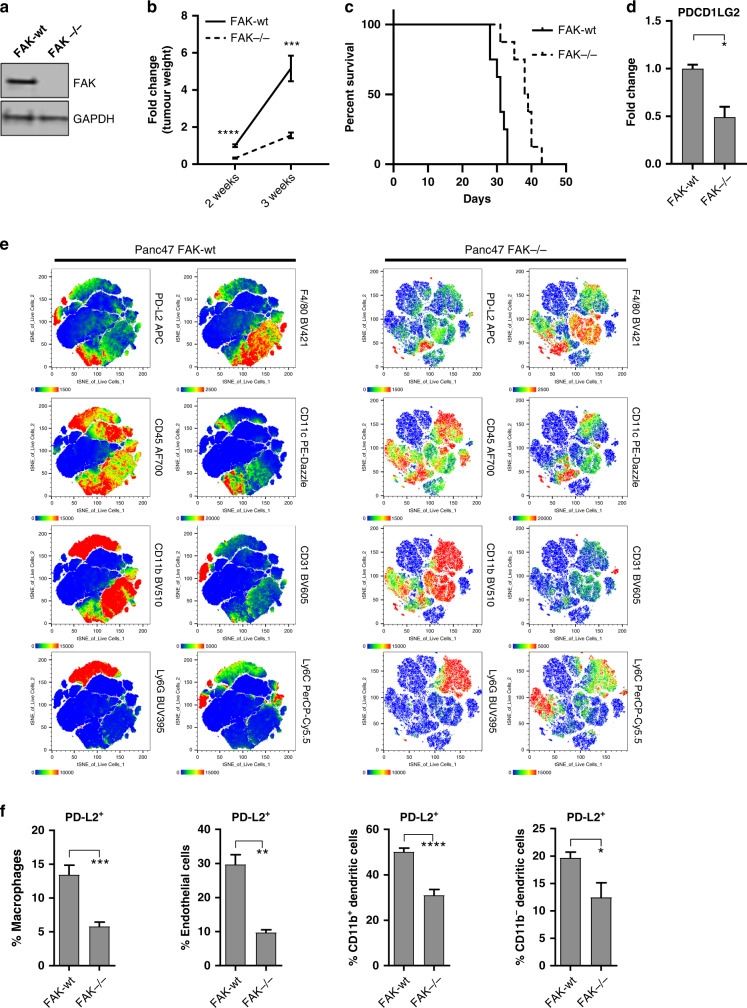
FAK promotes murine PDAC growth associated with increased stromal expression of PD-L2. **a** Representative western blot of Panc47 FAK-wt and FAK^−/−^ whole cell lysates probed with anti-FAK and anti-GAPDH antibodies. **b** Average weight of Panc47 FAK-wt and FAK^−/−^ tumours 2 and 3 weeks post-implantation of 0.5 × 10^6^ cells into the pancreas of C57BL/6 mice. *n* = 8 tumours per group. **c** Kaplan–Meier survival plot of C57BL/6 mice implanted with Panc47 FAK-wt and FAK^−/−^ tumours. *n* = 8 mice per group. Log-rank (Mantel-Cox) test, *p* = 0.0005; Gehan-Breslow-Wilcoxon test, *p* = 0.0015. **d** Nanostring gene expression analysis of RNA isolated from Panc47 FAK-wt and FAK^−/−^ tumours. *n* = 3 tumours per group. **e** t-sne map of flow cytometry data from Panc47 FAK-wt and Panc47 FAK^−/−^ tumours. Data generated from all live cells in a representative tumour. **f** Flow cytometry quantification of PD-L2 expression in Panc47 FAK-wt and FAK^−/−^ tumours. *n* = 9 tumours per group. Data represented as mean ± s.e.m. Two-tailed unpaired *t*-test, ****p ≤ 0.0001, ***p ≤ 0.001, **p ≤ 0.01, *p ≤ 0.05.

FAK activity is elevated in human PDAC [17], and inhibition of FAK function using small molecule kinase inhibitors or scaffolding inhibitors can impair PDAC growth [17, 27]. FAK kinase inhibitors can also sensitise murine PDAC to immunotherapies [17, 24]. We therefore set out to further define the mechanisms through which FAK contributes to the regulation of PDAC growth and immune evasion. We first used CRISPR-Cas9 gene editing to deplete *fak* expression in Panc47 cells, a cell line isolated from PDAC arising on *LSL-Kras*^*G12D/+*^*;LSL-Trp53*^*R172H/+*^*;Pdx-1-Cre* mice, and re-expressed wild-type FAK (FAK-wt) into Panc47 FAK^−/−^ cells (Fig. 1a). 0.5 × 10^6^ Panc47 FAK-wt or FAK^−/−^ cells were implanted into the pancreas of C57BL/6 mice, and the mice were culled either 2 or 3 weeks post-implantation. Tumours were harvested and weighed to determine the effects on tumour growth. FAK^−/−^ tumours were significantly smaller than FAK-wt tumours; however, both tumour types showed increased growth over time (Fig. 1b). Concomitant with impaired growth and in agreement with previously reported observations using a FAK inhibitor [17], FAK-depletion also resulted in a statistically significant increase in overall survival (Fig. 1c). Therefore, pancreatic cancer cell-intrinsic FAK signalling promotes PDAC growth.

To further explore FAK-dependent mechanisms of immune suppression in PDAC we next implanted 0.5 × 10^6^ Panc47 FAK-wt and FAK^−/−^ cells into the pancreas of C57BL/6 mice, sacrificed mice 3 weeks later, and prepared whole-tumour RNA extracts for gene expression analysis using Nanostring. These data identified a significant decrease in expression of the immune checkpoint ligand *pdcd1lg2* (PD-L2 gene) in Panc47 FAK^−/−^ tumours when compared to FAK-wt tumours (Fig. 1d), supporting our previous observations in murine skin squamous cell carcinoma (SCC) that treatment with the FAK inhibitor BI 853520 could decrease PD-L2 expression on multiple cell types within the TME, contributing towards the improved anti-tumour efficacy of FAK/immunotherapy combinations [24]. To better define which cell types within the PDAC TME expressed PD-L2, 0.5 × 10^6^ Panc47 FAK-wt and FAK^−/−^ cells were implanted into the pancreas of C57BL/6 mice and tumours were allowed to develop for 2 weeks. Mice were then sacrificed, tumours harvested and flow cytometry was used to identify cell populations expressing PD-L2. Initially, flow cytometry data were analysed using t-distributed stochastic neighbour embedding (tSNE) in order to identify cell-type markers associated with the expression of PD-L2 (Fig. 1e). These analyses suggested that endothelial cells, dendritic cells (DCs) and some macrophages were the predominant sources of PD-L2 expression. Conventional flow cytometry gating confirmed these observations (Supplementary Table 1, Supplementary Figs. 1 and 2) and a comparison of FAK-wt and FAK^−/−^ tumours identified that FAK-depletion results in a downregulation of PD-L2 expression on tumour-associated macrophages, endothelial cells, CD11b^+^ DCs and CD11b^−^ DCs (Fig. 1f). Therefore, FAK broadly regulates PD-L2 expression in the PDAC TME.

### PD-L2 is associated with poor patient survival in PDAC

**Fig. 2:**
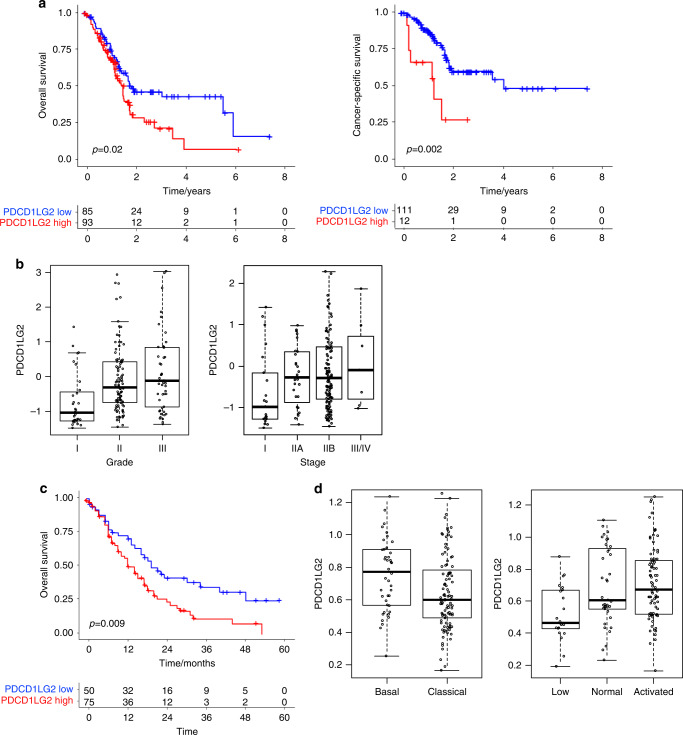
PD-L2 expression is associated with tumour grade, molecular subtype and poor patient survival in PDAC. **a** Kaplan–Meier plot of overall survival (left) and cancer-specific survival (right) in PDAC patients with low and high expression of PDCD1LG2. Source data: TCGA. **b** Left—PDCD1LG2 expression in grade I–III PDAC tumours. Source data: TCGA. Grade I vs III, *p* = 0.0001. right—PDCD1LG2 expression in stage I–IV PDAC tumours. Source data: TCGA. Grade I vs all other stages, *p* = 0.004. **c** Kaplan–Meier plot of survival probability in PDAC patients with low and high expression of PDCD1LG2. Source data: Moffit et al. **d** Left—PDCD1LG2 expression in Classical vs Basal PDAC tumour subtypes. Source data: Moffit et al. *p* = 0.01. right—PDCD1LG2 expression in low, normal and activated stromal PDAC tumour subtypes. Source data: Moffit et al. low vs activated *p* = 0.0005.

PD-L2 is one of two ligands for the immune checkpoint receptor Programmed Death Receptor 1 (PD-1), the other being PD-L1. In comparison to PD-L1, PD-L2 remains largely underinvestigated, especially in the context of pancreatic cancer. To address this, we examined two public transcriptomics datasets from human PDAC for which patient outcome and additional tumour characterisation are available [28, 29]. Analysis of data from The Cancer Genome Atlas (TCGA) identified that high PDCD1LG2 expression was associated with reduced overall and cancer-specific survival in patients with PDAC (Fig. 2a). In addition, subdividing tumours based on grade and clinical stage, two commonly used clinical classification systems, identified that PDCD1LG2 expression was significantly elevated in patients with PDAC tumours of advanced grade and clinical stage when compared with either early grade or stage tumours (Fig. 2b). To support these findings, we also performed a similar analysis using an independent dataset (GSE71729) from Moffitt et al. [29]. Again, elevated expression of PDCD1LG2 in primary PDAC was associated with poor outcome (Fig. 2c), validating findings from the TCGA dataset. Multiple gene expression studies have identified molecular subtypes of PDAC with biological and prognostic significance [29–31]. Based on their dataset, Moffitt et al. proposed two classification systems which were independently prognostic. One classification system identified PDAC tumours as either ‘basal-like’ or ‘classical’, and the other in which stromal subtypes were defined as ‘normal’ or ‘activated’. A further subset of PDAC was also described as having ‘low’ expression of stromal-associated genes. Analysis of PDCD1LG2 expression in these subtypes identified significantly elevated expression in ‘basal-like’ versus ‘classical’ tumours and in PDAC with an ‘activated’ stromal subtype versus those classified as ‘low’ (Fig. 2d). Both the ‘basal-like’ tumours and ‘activated’ stromal subtypes are associated with poorer survival, further supporting the conclusion that high PDCD1LG2 expression is a prognostic marker of poor clinical outcome in patients with PDAC.

### PD-L2 is predominantly expressed in the stroma of human PDAC

**Fig. 3:**
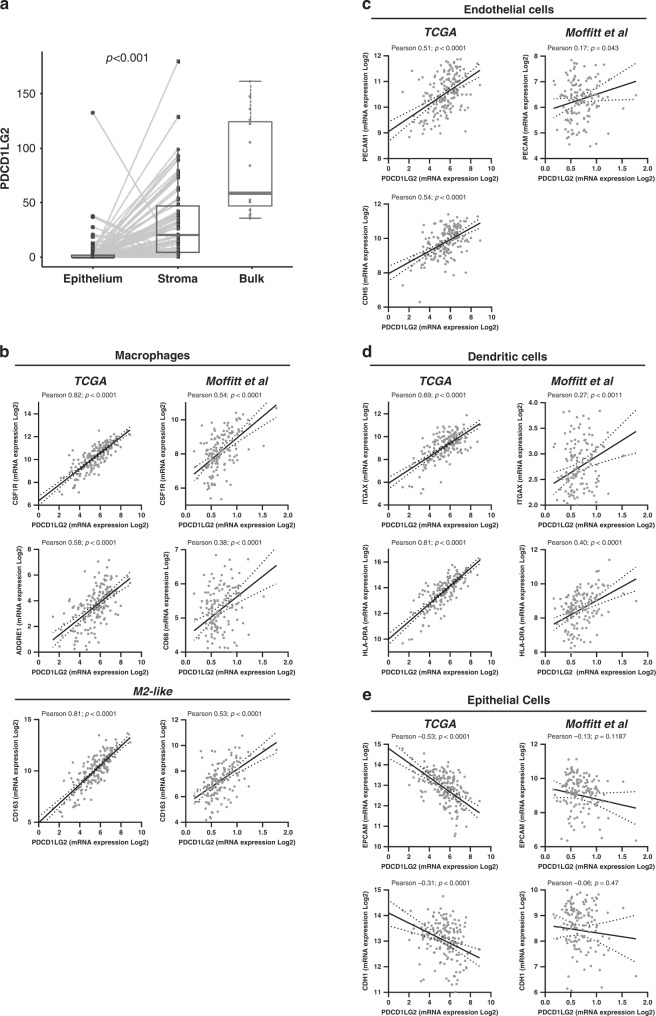
PD-L2 is predominantly expressed in in human PDAC stroma. **a** PDCD1LG2 expression in LCM separated RNASeq analysis of 66 pairs of Epithelium and Stroma and 15 bulk human PDAC [32]. **b**–**e** Co-expression of PDCD1LG2 and genes associated with immune cell subsets in TCGA [28] and Moffitt et al. [29] bulk RNAseq datasets from primary human PDAC.

Our finding identifying FAK-dependent expression of PD-L2 in a murine model of PDAC (Fig. 1) also suggested that PD-L2 was predominantly expressed in the stroma rather than neoplastic epithelial cells. To determine if this was also the case in human PDAC we first analysed a publicly available transcriptomics dataset representing 66 matched pairs of laser capture micro-dissected human PDAC epithelium and stroma e.g. fibroblast, myeloid, lymphoid, endothelial and other cell lineages [32]. Pairwise and absolute expression of PDCD1LG2 was significantly higher in stroma versus epithelium (Fig. 3a). To further validate that the expression pattern of PD-L2 observed in our mouse model was reflected in human PDAC, we next analysed both the TCGA and Moffitt et al. datasets in order to determine the correlation between PDCD1LG2 expression and gene markers associated with macrophages (CSF1R, ADGRE1, CD68, CD163), endothelial cells (CDH5, PECAM), dendritic cells (ITGAX, HLA-DRA) and epithelial cells (EPCAM, CDH1) (Fig. 3b–e). A positive correlation between PDCD1LG2 expression and genes associated with macrophages (including alternatively activated M2-like macrophages), endothelial cells and dendritic cells was identified in both transcriptomics datasets, while we also found a negative correlation between PDCD1LG2 expression and markers of epithelial cells. Thus, murine models of PDAC, such as we have used here, accurately recapitulate the expression pattern of PD-L2 in human PDAC, supporting their suitability for studies aimed at better understanding mechanisms of PD-L2 regulation.

### FAK-dependent expression of PD-L2 requires CD4^+^ T-cells

**Fig. 4:**
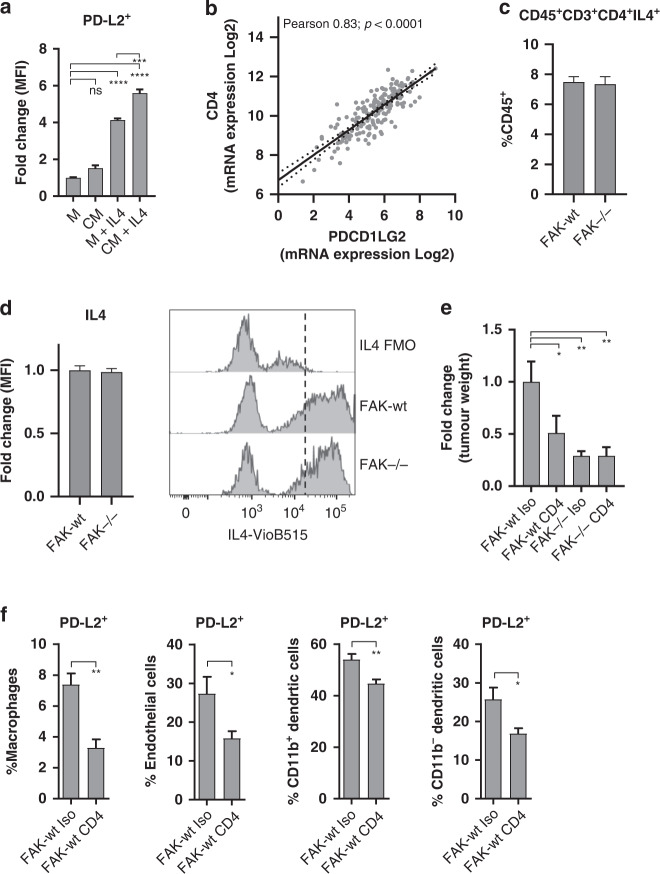
FAK-dependent expression of stromal PD-L2 requires CD4^+^ T cells. **a** Flow cytometry quantification of PD-L2 expression on bone-marrow derived macrophages cultured in normal growth media (M), FAK-wt conditioned media (CM) or M / CM supplemented with IL4. *n* = 4 per condition. Ordinary one-way ANOVA with Tukey’s multiple comparison. **b** Analysis of the correlation between CD4 and PDCD1LG2 gene expression in human PDAC RNAseq data (TCGA, 184 samples). **c** Flow cytometry quantification of CD45^+^CD3^+^CD4^+^IL4^+^ cells as a percentage of CD45^+^ cells in Panc47 FAK-wt and FAK^−/−^ tumours. *n* = 6 per condition. **d** Left—Flow cytometry quantification of the median fluorescence intensity of IL4 expression in CD45^+^CD3^+^CD4^+^IL4^+^ cells from Panc47 FAK-wt and FAK^−/−^ tumours. Data represented as a fold-change relative to FAK-wt. *n* = 6 per condition. Right—representative histogram showing IL4 expression compared to FMO control. **e** Fold-change in average weight of Panc47 FAK-wt and FAK^−/−^ tumours following treatment with either isotype control or anti-CD4 antibodies. *n* = 3, one-way ANOVA Fisher’s LSD. **f** Flow cytometry quantification of PD-L2 expression in Panc47 FAK-wt tumours following treatment with either isotype control antibody or anti-CD4 T-cell depleting antibody. *n* = 6, two-tailed unpaired *t*-test. All data represented as mean ± s.e.m. ****p ≤ 0.0001, ***p ≤ 0.001, **p ≤ 0.01, *p ≤ 0.05.

Our observation that FAK-depletion in pancreatic cancer cells could impact the expression of PD-L2 within the PDAC TME led us to hypothesise that this may be mediated via a paracrine signalling mechanism. We therefore generated monocyte-derived macrophages and treated these with either normal cell culture media (M), M + interleukin-4 (IL4), Panc47 FAK-wt conditioned media (CM) or Panc47 FAK-wt CM + IL4 (Fig. 4a). IL4 has previously been shown to potently induce the expression of PD-L2 and was therefore initially used as a control [33]. CM media alone was not sufficient to promote the expression of PD-L2 on monocyte-derived macrophages. However, when used in combination with IL4, CM from FAK-wt cells amplified the expression of PD-L2 when compared to IL4 alone. Thus, FAK-wt cells secrete an unknown factor that can enhance the action of IL4 in promoting the expression of PD-L2.

Based on these findings, we next sought to identify whether there was a source of IL4 within PDAC tumours and whether this may also be regulated by FAK. Th2 polarised CD4^+^ T-cells secrete IL4, which can robustly induce PD-L2 expression in other cell types [33]. Furthermore, the Th2: Th1 ratio in pancreatic tumours has been linked to patient prognosis [15], and multiple lines of evidence support a role for CD4^+^ T-cells in promoting pancreatic tumorigenesis [13–15, 34, 35]. Therefore, we hypothesised that CD4^+^ T-cells may be required for FAK-dependent induction of PD-L2 expression in the PDAC TME. To support this hypothesis, we first determined whether there was evidence of a link between CD4^+^ T-cells and PD-L2 expression in human PDAC. Analysis of publicly available bulk RNAseq data from human PDAC (TCGA [28]) identified a statistically significant positive correlation between *CD4* expression and *PDCD1LG2* expression (Fig. 4b), implying that these genes are co-expressed. We next implanted 0.5 × 10^6^ Panc47 FAK-wt and FAK^−/−^ cells into the pancreas of C57BL/6 mice, culled mice 2 weeks post-implantation, and processed tumours for flow cytometry analysis to identify IL4^+^ CD4^+^ T-cells. CD45^+^CD3^+^CD4^+^IL4^+^ T-cells were present in both FAK-wt and FAK^−/−^ tumours at similar levels (Fig. 4c), and IL4 expression by these cells was also similar (Fig. 4d). Therefore, FAK does not regulate CD45^+^CD3^+^CD4^+^IL4^+^ T-cell numbers or the phenotype of these cells with regards to IL4 expression. To formally test whether CD4^+^ T-cells were important in promoting tumour growth and driving PD-L2 expression, 0.5 × 10^6^ Panc47 FAK-wt cells were implanted into the pancreas of C57BL/6 mice and mice treated with either isotype control or anti-CD4 depleting antibodies. Mice were culled 2 weeks post-implantation and tumours were weighed and processed for analysis by flow cytometry. Treatment with an anti-CD4 depleting antibody resulted in a significant reduction in FAK-wt tumour growth but had no effect on the growth of FAK^−/−^ tumours (Fig. 4e). CD4^+^ T-cell depletion also resulted in the downregulation of PD-L2 expression on tumour-associated macrophages, endothelial cells, and DCs (Fig. 4f). Therefore, CD4^+^ T-cells play an important role in promoting the expression of PD-L2 on multiple cell types within the PDAC TME.

### FAK regulates IL6 to amplify IL4-dependent PD-L2 expression

**Fig. 5:**
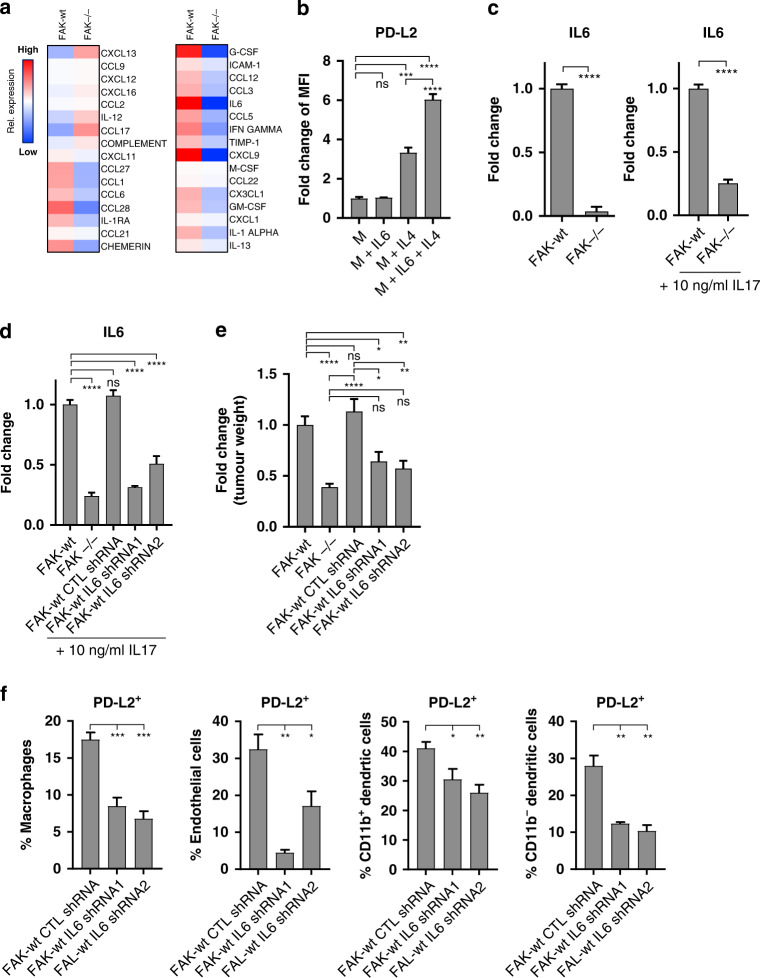
FAK-dependent expression of IL6 amplifies IL4-dependent PD-L2 expression. **a** Quantitative analysis of chemokines / cytokines present in media conditioned by either Panc47 FAK-wt or Panc47 FAK^−/−^ cells for 48 h. **b** Flow cytometry quantification of PD-L2 expression on bone-marrow derived macrophages cultured in normal growth media (M), M + IL6, M + IL4 and M + IL6 + IL4. Data represented as fold-change in median fluorescence intensity relative of M alone. *n* = 3 per condition, ordinary one-way ANOVA with Tukey’s multiple comparison. **c** ELISA quantification of IL6 in media conditioned by either Panc47 FAK-wt or Panc47 FAK^−/−^ cells untreated or stimulated with IL17. *n* = 3, two-tailed unpaired *t*-test. **d** ELISA quantification of IL6 in media conditioned by either Panc47 FAK-wt (*n* = 7), FAK^−/−^ (*n* = 7), FAK-wt control shRNA (*n* = 7), FAK-wt IL6 shRNA1 (*n* = 6) and FAK-wt IL6 shRNA2 cells (*n* = 6). One-way ANOVA with Dunnett’s multiple comparison. **e** Fold-change in the average weight of Panc47 FAK-wt (*n* = 14), FAK^−/−^ (*n* = 11), FAK-wt control shRNA (*n* = 4), FAK-wt IL6 shRNA1 (*n* = 6) and FAK-wt IL6 shRNA2 (*n* = 6) tumours 2 weeks post-implantation of 0.5 × 10^6^ cells into the pancreas of C57BL/6 mice. Ordinary one-way ANOVA with Tukey’s multiple comparison. **f** Flow cytometry quantification of PD-L2 expression in Panc47 FAK-wt CTL shRNA (*n* = 7), FAK-wt IL6 shRNA1 (*n* = 3) and FAK-wt IL6 shRNA2 (*n* = 3) tumours. Ordinary one-way ANOVA with Tukey’s multiple comparison. All data represented as mean ± s.e.m. ****p ≤ 0.0001, ***p ≤ 0.001, **p ≤ 0.01, *p ≤ 0.05.

To identify candidate soluble factors preferentially secreted by Panc47 FAK-wt cells with the potential to amplify IL4-induced PD-L2 expression, cell culture media was conditioned using FAK-wt or FAK^−/−^ cells for 48 h and forward-phase chemokine/cytokine arrays used to profile their secretome (Fig. 5a). In general, broad reprogramming of chemokine and cytokine secretion was observed in response to FAK-depletion. From the list of cytokines/chemokines upregulated in FAK-wt cells, we decided to focus on interleukin-6 (IL6), as this has previously been associated with the expression of PD-L2 [36]. We therefore generated monocyte-derived macrophages and treated these with either normal cell culture media (M), M + IL6, M + IL4 or M + IL6 + IL4 (Fig. 5b). IL6 alone did not induce PD-L2 expression. However, the combination of IL6 + IL4 resulted in a significant increase in PD-L2 expression when compared to either IL4 or IL6 alone, identifying IL6 as a candidate factor secreted by FAK-wt cells that could enhance IL4-dependent expression of PD-L2. Anti-IL6 ELISA assays further confirmed FAK-dependent regulation of IL6 both under basal culture conditions and when cells were treated with interleukin-17 (IL17), a strong stimulus of IL6 expression commonly found in the pancreatic TME [37] (Fig. 5c). To ensure that regulation of IL6 by FAK was not exclusive to Panc47 FAK-wt and FAK^−/−^ cells, further FAK^−/−^ CRISPR clones and their FAK-wt reconstituted counterparts were also tested for IL6 secretion using chemokine/cytokine forward-phase arrays (Supplementary Fig. 3). In all CRISPR clones tested, FAK-depletion resulted in a reduction of IL6 secretion. Many of FAK’s cellular functions are dependent on its kinase activity, and a number of FAK kinase inhibitors are now in Phase-I/II clinical trials [38–42]. Therefore, to determine whether FAK’s regulation of IL6 was dependent on its kinase activity, Panc47 FAK-wt cells were treated with BI 853520, a highly potent and specific FAK inhibitor currently in clinical development [24, 40]. Increasing concentrations of BI 853520 identified that a concentration of 100 nM was sufficient for maximal inhibition of FAK phosphorylation on tyrosine 397 (Supplementary Fig. 4A), the autophosphorylation site commonly used as a surrogate readout of FAK kinase activity. Treatment of Panc47 FAK-wt and FAK^−/−^ cells with 100 nM BI 853520 resulted in a significant reduction in the levels of IL6 in FAK-wt CM, but had no effect on the levels of IL6 in FAK^−/−^ CM (Supplementary Fig. 4B). Thus, FAK kinase activity likely plays an important role in regulating the expression/secretion of IL6. Previous findings, using a mouse model of SCC, have also identified an important role for nuclear FAK in regulating chemokine and cytokine expression [26]. To determine whether regulation of IL6 by FAK in Panc47 cells was also dependent on nuclear FAK, a FAK mutant in which nuclear targeting was impaired (FAK-NLS) was re-expressed into Panc47 FAK^−/−^ cells (Supplementary Fig. 5A). Panc47 FAK-wt, FAK^−/−^ and FAK-NLS cells were then used to condition growth media for 48 h, and an anti-IL6 ELISA used to measure IL6 secretion. In contrast to our previous findings, IL6 secretion was not dependent on FAK nuclear translocation (Supplementary Fig. 5B).

Having established that CD4^+^ T-cells promote PD-L2 expression in Panc47 FAK-wt tumours and that IL6 present in FAK-wt CM can enhance IL4-dependent expression of PD-L2, we next sought to determine whether IL6 secreted by PDAC cells was also important for Panc47 tumour growth and PD-L2 expression in vivo. Panc47 FAK-wt cells were transduced using lentiviral infection with plasmids encoding shRNA targeting IL6 or a control non-targeting shRNA. An anti-IL6 ELISA confirmed knockdown of IL6 expression using two different IL6-specific shRNAs (FAK-wt shRNA1 and shRNA2) and no effect on IL6 secretion using a control non-targeting shRNA (FAK-wt CTL shRNA) (Fig. 5d). Western blotting confirmed that IL6-depletion had no effect on FAK expression and activation (Supplementary Fig. 6). 0.5 × 10^6^ Panc47 FAK-wt, FAK^−/−^, FAK-wt CTL shRNA, FAK-wt IL6 shRNA1, or FAK-wt shRNA2 cells were implanted into the pancreas of C57BL/6 mice, mice sacrificed 2 weeks post-implantation and tumours weighed (Fig. 5e). IL6-depletion in FAK-wt cells resulted in a significant reduction in tumour growth when compared to either FAK-wt or FAK-wt shRNA CTL tumours. Thus, FAK-dependent expression of IL6 promotes PDAC growth. We next sought to determine whether IL6 secreted by FAK-wt cells was also required for PD-L2 expression in Panc47 tumours. 0.5 × 10^6^ Panc47 FAK-wt CTL shRNA, FAK-wt IL6 shRNA1 or FAK-wt IL6 shRNA2 cells were implanted into the pancreas of C57BL/6 mice, mice sacrificed 2 weeks later, and tumours processed for analysis using flow cytometry. IL6-depletion resulted in the downregulation of PD-L2 expression on tumour-associated macrophages, endothelial cells, and DCs (Fig. 5f), suggesting that FAK-dependent expression of IL6 promotes PD-L2 expression in the PDAC TME.

## Discussion

**Fig. 6:**
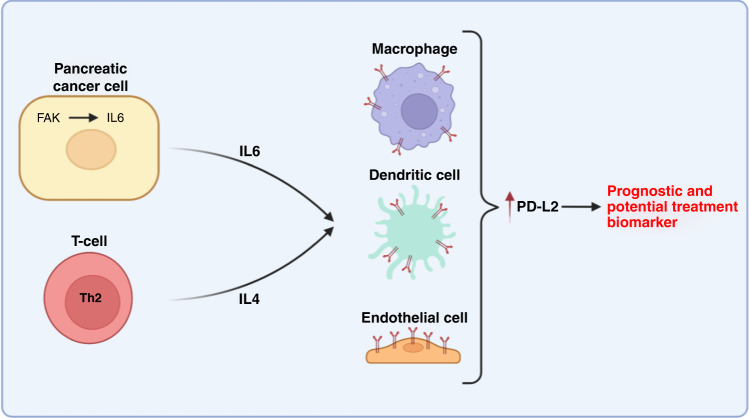
Model. Graphical summary of proposed mechanism through which FAK-IL6 signaling amplifies PD-L2 expression in the PDAC stroma. Image created using BioRender.

In this study, we show that FAK expressed in *kras* mutant murine pancreatic cancer cells regulates the expression of IL6, which acts to amplify IL4-dependent expression of the immune checkpoint ligand PD-L2 within the PDAC TME (Fig. 6). We further identify that elevated PD-L2 expression in human PDAC is associated with tumour grade, clinical stage, molecular subtype and poor patient prognosis. These findings provide new insight into mechanisms through which FAK promotes immune escape in PDAC.

To date, therapies targeting the PD-1/PD-L1 pathway have shown little clinical activity in the treatment of PDAC, with the potential exception of microsatellite unstable tumours [4, 43]. However, this does not necessarily mean that these pathways have no role to play in the development of at least a subset of PDAC through promoting immune evasion or that they will not have therapeutic value in the treatment of some patients with PDAC. Rather, it supports the hypothesis that multiple immune evasion mechanisms are likely active in PDAC and that the exact nature of these may vary between patients. Therefore, simultaneous targeting of multiple immune evasion mechanisms may be required to unlock effective anti-tumour immunity against PDAC. In this context, FAK is emerging as a promising target; previous studies have shown that FAK inhibition using a small molecule inhibitor or genetic ablation can regulate a variety of immuno-suppressive cell types within tumours, including in PDAC [17, 26]. Our data imply that mechanisms of FAK-dependent immune suppression extend beyond the control of immune cell recruitment to include the regulation of molecular pathways associated with immune evasion and patient outcome. This observation is supported by our previously published work showing broad downregulation of PD-L2 expression in murine skin SCC tumours in response to treatment with a FAK inhibitor [24]. Thus PD-L2 may be a common target of FAK-mediated immune suppression, perhaps via a conserved mechanism such as that we describe here. Multiple lines of evidence support the conclusion that PD-L2 contributes to an immuno-suppressive TME. In a mouse model of pancreatic cancer, PD-L2 blockade on DCs resulted in activation of CD8^+^ T-cells and suppression of metastasis [11]. PD-L2 blockade on macrophages can inhibit cytotoxic T-cell proliferation [44], and endothelial cell expression of PD-L2 has been reported to regulate CD8^+^ T-cell activation [45]. We have also previously shown that FAK-dependent modulation of PD-L2 contributes toward the enhanced anti-tumour efficacy of a FAK inhibitor in combination with an agonistic antibody targeting the T-cell co-stimulatory receptor OX40 [24]. This combination stimulated anti-tumour immunity against both murine skin SCC and pancreatic tumours. Therefore, we propose that PD-L2 represents an additional component of a multifaceted immune evasion program regulated by FAK in pancreatic cancer and that high expression of PD-L2 may also represent a potential biomarker for the identification of patients more likely to benefit from treatment with a FAK inhibitor. While treatment with a FAK inhibitor may lead to a reduction in PD-L2 expression within the TME, we did not identify a role for FAK in regulating PD-L1 (data not shown). Therefore, pre-clinical and clinical testing of FAK inhibitors in combination with anti-PD-1 or anti-PD-L1 targeted therapies may still offer potential benefits, such as has been reported previously [17] and is currently being tested in the clinic (clinicaltrials.gov NCT02758587, NCT02546531, NCT03727880). A recent meta-analysis of response efficacy to PD-1 and PD-L1 inhibitors across cancer types identified that targeting PD-1, especially in combination with chemotherapy, can result in improved response rates over that of similar PD-L1 combinations [46]. Such differences in activity may at least in part be due to PD-L2, which we show here to be regulated by FAK. Therefore, it is not clear at this stage whether there would be any difference in efficacy when combining FAK inhibitors with anti-PD-1 over anti-PD-L1, and such comparisons have not been reported to date.

Our data identified an important role for FAK-dependent expression of IL6 in amplifying IL4-induced expression of PD-L2. IL6 is a pro-inflammatory cytokine upregulated in a number of cancers, including breast [47], prostate [48], endometrial [49], renal cell carcinoma [50], oral squamous cell carcinoma [51], multiple myeloma [52], colorectal cancer [53] and pancreatic cancer [54]. It has previously been linked to promoting Th2 differentiation of CD4^+^ T-cells and shifting the Th1/Th2 balance in favour of Th2 [55, 56]. While we did not identify any impact of FAK loss on Th2 cells, our data suggest that FAK-dependent expression of IL6 may act in concert with IL4 secreted by Th2 cells to promote PD-L2 expression. IL6 overexpression has also been reported to promote Th17 differentiation of CD4^+^ T-cells with anti-tumour activity in a transplantable murine model of pancreatic cancer [57]. These conflicting findings suggest that IL6 function may be context-dependent, perhaps influenced by the relative levels of other cytokines within the TME. In PDAC, increased circulating levels of IL6 have been linked to tumour progression through modulating the TME and are generally associated with poorer clinical outcome [18, 58, 59]. In a murine model of pancreatic cancer, inhibition of IL6 in combination with inhibition of PD-L1 resulted in increased infiltration of effector CD8^+^ T-cells into tumours and impaired tumour growth [60]. Therefore, the majority of evidence supports the role of IL6 in promoting PDAC development and resistance to therapy. As a consequence, IL6 has emerged as a potential therapeutic target in pancreatic cancer, and a phase II clinical trial is underway aimed at evaluating the anti-tumour efficacy of tocilizumab, an anti-IL6 receptor antibody, in patients with unresectable pancreatic cancer (clinical trials.gov NCT02767557). Our findings suggest that IL6 may represent an important mechanism through which FAK regulates PDAC development and response to therapy, potentially contributing toward the enhanced anti-tumour activity of FAK inhibitors in combination with immunotherapies [17, 24]. These conclusions are based on the use of syngeneic orthotopic mouse models of PDAC, which, while offering a number of advantages for such mechanistic studies, also have their limitations. Therefore, complementary studies using genetically engineered mouse models of PDAC that more faithfully recapitulate the stages of PDAC development and therapy response would represent a logical next step in further developing our understanding of FAK-regulated IL6 in PDAC biology and therapeutic response.

Overall, these data support the continued exploration of FAK as a potential therapeutic target for the treatment of pancreatic cancer. Collectively, our findings and that of others [17, 27] suggest that FAK inhibition in murine models of PDAC can have wide-ranging effects on the PDAC TME and mechanisms of immune suppression. However, available data from Phase-I clinical trials indicates only modest anti-tumour activity when FAK inhibitors are used as a monotherapy [38, 39, 61]. Thus, future efforts should focus on understanding how to utilise FAK inhibitors and their immunomodulatory potential as part of rational drug combinations developed through a detailed understanding of FAK biology in PDAC and other cancers.

## Material and methods

### Materials

BI 853520 was provided by Boehringer Ingelheim GmbH. All recombinant mouse cytokines (IL4, IL6 and IL17) were purchased from Biolegend and used at a final concentration of 10 ng/ml. All flow cytometry antibodies used are listed in Supplementary Table 1.

### Cell lines

The Panc47 and Panc117 cell lines were a generous gift from Dr Jen Morton (CRUK Beatson Institute, Glasgow, UK). These cell lines were originally derived from PDAC arising on *LSL-Kras*^*G12D/+*^*;LSL-Trp53*^*R172H/+*^*; Pdx-1 Cre (KPC)* mice. All cell lines were cultured at 37 °C / 5% CO_2_ in Dulbecco’s Minimum Essential Medium—high glucose (Sigma) supplemented with 10% foetal bovine serum (Life Technologies). Cells were pathogen tested in September 2016 using the ImpactIII test (Idex Bioresearch) and were negative for all pathogens. Cell lines are routinely tested for mycoplasma every 2–3 months in-house and have never been found to be mycoplasma positive. Cell lines are cultured for no more than 3 months following freeze-thawing.

### CRISPR-Cas9

Type II CRISPR/Cas9 genome editing technology was used to deplete FAK expression in Panc47 and Panc117 cells as described in the protocol published by Ran et al. [62]. Briefly, guide RNAs (gFAK4: Forward oligo: p5’-CAC CGT TAC TCT AAT ACT TCA TAG T -3’; Reverse oligo: p5’-AAA CAC TAT GAA GTA TTA GAG TAA C-3; gFAK6: Forward oligo: p5’-CAC CGC ATA GTT GGA CTT CTT CTC T-3’; Reverse Oligo: p5’-AAA CAG AGA AGA AGT CCA ACT ATG C-3’) were cloned into the target vector pSPCas9(BB)-2A-GFP (PX458). To generate FAK-depleted Panc47 cell clones, cells were transfected with the expression plasmids containing either the gFAK4 or gFAK6 guide sequences using lipofectamine® 2000 (ThermoFisher Scientific). 7 days post-transfection, cells positive for GFP expression were single-cell sorted using a FACSAria II (BD Biosciences) into 96-well plates containing normal pancreatic culture media supplemented with Penicillin-Streptomycin (Gibco Life Technologies 10,000 U/mL, diluted 1:100). Resulting cell colonies were tested for successful depletion of FAK expression using anti-FAK western blotting. FAK-wt and FAK-NLS were re-expressed into individual Panc47 and Panc117 FAK−/− clones using retroviral transduction and selection with 0.25 mg/ml hygromycin.

### shRNA

Panc47-1 wt IL6 knockdown cells were generated as previously described [26] by lentiviral transduction of pLKO IL6 shRNA contructs (TRCN0000067548, TRCN0000067549, TRCN0000067550, TRCN0000067551, TRCN0000067552 (*Dharmacon*)) or non-targeting control pLKO-NTCO and selection with 2 μg/ml puromycin.

### Analysis of publicly available human PDAC transcriptomics datasets

Gene expression data was downloaded from cBioportal (https://cancerdiscovery.aacrjournals.org/content/2/5/401.abstract) and NCBI GEO (https://academic.oup.com/nar/article/41/D1/D991/1067995) from the two largest published pancreatic cancer transcriptomic studies [28, 29]. Comprehensive survival analysis to split cohorts into low and high groups by all possible (n-1) cut-points was performed using the survivALL R package (Pearce 2017—https://www.biorxiv.org/content/10.1101/208660v2). Multiple cut-points associated high PDCD1LG2 levels with poor outcomes, the most significant were plotted as Kaplan–Meier survival curves.

### Generation of bone-marrow-derived macrophages (BMDMs)

Bilateral tibias and femurs dissected from C57BL/6 mice were flushed with 5 ml of DMEM supplemented with 10% FBS and 1% Penicillin/Streptomycin into a 50 ml falcon tube, washed in a medium once and filtered through a 70 μm cell strainer. Cells were seeded at 1 × 10^6^ per well in a six-well plate and cultured in 2 ml of DMEM with 10% FBS and 25 ng/ml recombinant mouse M-CSF for 7 days. BMDMs were then washed with PBS followed by replacement with fresh media containing recombinant mouse cytokines or conditioned media from pancreatic cancer cells. BMDMs were cultured for a further 24 h, washed with PBS and harvested using non-enzymatic dissociation buffer, stained with fluorescently conjugated antibodies and analysed by Flow cytometry as described below.

### Immunoblotting

Cell lysates (10–20 μg protein, as measured by Micro BCA Protein Assay kit (Pierce)) were supplemented with SDS sample buffer (Tris, pH 6.8, 20% glycerol, 5% SDS, β-mercaptoethanol, and bromophenol blue), separated by SDS–PAGE, transferred to nitrocellulose and immunoblotted with specific antibodies [anti-FAK antibody (1:1000, clone 4.47; Millipore), anti-FAK pY397 antibody (1:1000, Cell Signalling Technology) or anti-α-tubulin (clone DN1A, Cell Signalling Technology). Fluorescent detection was carried out upon incubation with DyLight™ 680/ 800 Conjugated secondary antibodies (1:15,000, Cell Signalling Technology) in a LI-COR Odyssey CLx scanner (LI-COR Biosciences).

### Enzyme-linked immunosorbent assay (ELISA)

Conditioned media was collected after 48 h incubation, and ELISA assay was carried out using a mouse IL6 DuoSet ELISA kit (R&D systems) according to the manufacturer’s instructions. Cells were lysed in RIPA buffer, and protein concentrations were determined in order to normalise ELISA results.

### Forward-phase protein arrays (FPPA)

Conditioned media was collected after 48 h incubation. Microarrays were generated using the in-house Aushon BioSystems’ 2470 array printing platform. Microarrays were blocked for 1 h with SuperG™ Blocking Buffer (Grace Bio Labs) at room temperature on a rocker. Media from samples were centrifuged at 1000 × *g* for 5 min at 4 °C. Supernatants were added to microarrays for 12 h at 4 °C. Microarrays were washed three times for 5 min in TBST and blocked for 10 min with SuperG™ Blocking Buffer at room temperature on an orbital shaker, then washed again washed three times for 5 min in TBST. Detection antibodies (1:500 antibody diluted in 5% BSA/PBST, 1% SuperG™ Blocking Buffer) mixtures were made in plates, and 2 μl of each antibody was applied to each well of the microarrays. Microarrays were clamped, and 50 μl of each antibody was added to corresponding microarray wells. Microarrays were incubated for 1 h on a flat surface. Clamps were removed, and microarrays were washed three times for 5 min in TBST. Microarrays were then blocked for 10 min with SuperG™ Blocking Buffer at room temperature on a rocker and again washed three times for 5 min in PBST. 3 ml of IRDye® 800CW Streptavidin (LI-COR Biosciences) diluted 1 in 5000 in PBST supplemented with 5% BSA, 1% SuperG™ Blocking Buffer. Microarrays were covered and incubated on a rocker at room temperature for 45 min then washed for 5 min, three times in PBST followed by three 5 min PBS washes and then washed with distilled water. Microarrays were dried and then scanned on the InnoScan 710 high-resolution Microarray scanner (Innopsys Life Sciences). Data were normalised for protein concentration and background fluorescence in Microsoft Excel. Data were median centred and subjected to unsupervised agglomerative hierarchical clustering on the basis of Euclidean distance computed with a complete-linkage matrix using Cluster 3.0 [63]. Clustering results were visualised using Java TreeView [64].

### Orthotopic implantation of cancer cells into the pancreas

Female C57BL/6 mice (Envigo, UK) were supplied as age-matched, 5-week-old females and isolated for 1 week after delivery. All experiments had University of Edinburgh ethical approval and were carried out in accordance with the United Kingdom Animal Scientific Procedures Act (1986) under Home Office Project License number PP7510272. Mice were randomly allocated to groups prior to surgery.

Mice were anaethestised with inhalational isoflurane anaesthetic in oxygen, and received perioperative analgesia: buprenorphine (Vetergesic, 0.1 mg/kg s.c) and carprofen (Rimadyl,10 mg/kg s.c) and also post-surgery, once daily for 48 h. Cell lines were propagated to sub-confluency to ensure they were in their exponential growth phase. Once detached from the flask and washed with PBS, 0.5 × 10^6^ cells of the appropriate cell line were suspended in growth factor reduced matrigel basement membrane matrix (Scientific Laboratory Supplies Ltd.), at a concentration of 0.5 × 10^6^ cells in 25 μl. Using an aseptic technique, a 3 mm skin incision was made in the left lateral flank and lateral abdominal muscles in order to visualise the pancreas. 0.5 × 10^6^ cells in 25 μl matrigel were injected into the pancreas in a sterile manner. The abdominal wall was closed with Polyglactin 910 (Vicryl, 2 M, Henryschein), with a single cruciate suture. The skin was closed with skin clips. Mice were monitored in a heat box set to 37 °C post-surgery for 1 h. Mice were closely monitored daily with twice weekly weight checks following implantation. If any single terminal symptom caused by pancreatic tumour growth, including weight loss equal to or exceeding 10% of the starting weight, signs of abdominal pain or abdominal distension became apparent, the animal was humanely euthanised. After two weeks, the animals were culled (cervical dislocation), and the pancreatic tumours were harvested for analysis. Tumour weights were recorded and agreed upon by two observers.

### CD4^+^ T-cell depletion

Anti-mouse CD4 depleting antibody (GK1.5, ATCC TIB-207) and isotype control were purchased from BioXcell. Mice were treated with 100 μg of antibody administered by intraperitoneal injection for 3 consecutive days, followed by a rest period of 3 days. Following this, cells were surgically implanted into the pancreas and T-cell depletion was maintained by further administration of 100 μg depleting antibody at 3-day intervals for the remainder of the experiment. Mice were culled two weeks after surgery and pancreatic tumours were harvested for analysis as described above.

### FACS analysis

Tumours established following intra-pancreatic injections of cells into mice were removed on day 14 into DMEM (Sigma–Aldrich). Tumour tissue was mashed using a scalpel and re-suspended in DMEM (Sigma–Aldrich) supplemented with 2 mg/ml collagenase D (Roche) and 40 units/ml DNase1 (Roche). Samples were incubated for 30 min at 37 °C, 5% CO2 on an orbital shaker set at 120 rpm, and then pelleted by centrifugation at 1300 rpm for 5 min at 4 °C. Samples were re-suspended in 5 ml of red blood cell lysis buffer (Pharm Lysis Buffer, Becton Dickinson) for 10 min at 37 °C, pelleted by centrifugation at 1300 rpm for 5 min at 4 °C, re-suspended in PBS and mashed through a 70 μm cell strainer using the plunger from a 5 ml syringe. The cell strainer was further washed with PBS. The resulting single-cell suspension was pelleted by centrifugation at 1300 rpm for 5 min at 4 °C and re-suspended in PBS. This step was repeated twice. The resulting cell pellet was re-suspended in PBS containing Zombie NIR viability dye [1:1000 dilution (BioLegend)] and incubated at 4 °C for 30 min, then pelleted by centrifugation at 1300 rpm for 5 min at 4 °C. Cells were re-suspended in FACS buffer and pelleted by centrifugation at 1300 rpm for 5 min at 4 °C. This step was repeated twice. Cell pellets were re-suspended in 100 μl of Fc block [1:200 dilution of Fc antibody (eBioscience) in FACS buffer] and incubated for 15 min. 100ul of antibody mixture [diluted in FACS buffer (antibody details listed in supplementary tables 1 and 2)] was added to each well and the samples incubated for 30 min in the dark. The cells were then pelleted by centrifugation at 1300 rpm for 5 min at 4 °C and washed twice with FACS buffer as above. Finally, cells were re-suspended in FACS buffer and analysed using a BD Fortessa. Data analysis was performed using FlowJo software. Statistics and graphs were calculated using Prism (GraphPad). For flow cytometry analysis of cell lines, growth media was removed, and cells were washed twice in PBS. Adhered cells were dissociated from tissue culture flasks by incubating them in enzyme-free cell dissociation solution (Millipore) for 10 min at 37 °C, 5% CO_2_, and then scraping with a cell scraper. Cells were pelleted by centrifugation at 1300 rpm for 5 min at 4 °C and washed with PBS. This step was repeated twice. Cells were then re-suspended in viability dye and stained as above. For flow cytometry analysis of intracellular cytokines, the cell suspension was incubated with a protein transport inhibitor cocktail (eBioscience) for an hour prior to staining. After staining with viability and surface markers was completed as described above, the cell suspension was permeabilised using the Cyto-Fast Fix/Perm buffer set (Biolegend) and subsequently incubated with anti-IL4 antibody for 30 min in the dark. Cells were then washed and prepared for flow cytometry as described above.

### Nanostring analyses

RNA extracts were obtained using an RNeasy kit (Qiagen), following the manufacturer’s instructions. One hundred nanograms of RNA was analysed using a mouse nanostring PanCancer Immune Profiling panel as per the manufacturer's instructions. Hybridisation was performed for 18 h at 65 °C, and samples were processed using the nanostring prep station set on high sensitivity. Images were analysed at a maximum (555 fields of view). Data were normalised using nSolver 4.0 software.

### Statistics

Statistical analysis was carried out using GraphPad Prism8 for Windows (GraphPad Software). All error bars on graphs represent the standard error of the mean (sem). Statistical tests are detailed in the figure legends. n numbers provided for each experiment in the figure legends represent biological replicates. Sample sizes for in vivo experiments were not predetermined as data were accumulated on a rolling basis and analysis ongoing during this period.

## Supplementary information

Supplementary Material

checklist

## Data Availability

Gene expression data were downloaded from cBioportal (https://cancerdiscovery.aacrjournals.org/content/2/5/401.abstract) and NCBI GEO (https://academic.oup.com/nar/article/41/D1/D991/1067995) as detailed in materials and methods.
