# Supplementary material for: FAK promotes stromal PD-L2 expression associated with poor survival in pancreatic cancer
Source: Br J Cancer. 2022 Sep 22;127(10):1893–905. doi: 10.1038/s41416-022-01966-5 (PMC9643373; doi:10.1038/s41416-022-01966-5)
Supplement: Supplementary file 1 — Supplementary Material [file 41416_2022_1966_MOESM1_ESM.docx]

| **Marker** | **Fluorophore** | **Supplier** | **Clone** |
| --- | --- | --- | --- |
| **Ly6G** | **BUV395** | **BD Horizon** | **1A8** |
| **F480** | **BV421** | **Biolegend** | **BM8** |
| **CD11b** | **BV510** | **Biolegend** | **M1/70** |
| **CD31** | **BV605** | **Biolegend** | **390** |
| **Ly6C** | **PerCP/Cy5.5** | **Biolegend** | **HK1.4** |
| **CD11c** | **PE-Dazzle** | **Biolegend** | **N418** |
| **PD-L2** | **APC** | **Biolegend** | **TY25** |
| **CD45** | **AF700** | **Biolegend** | **30-F11** |
| **Viability** | **Zombie NIR** | **Biolegend** | **Cat# 423105** |
| **CD4** | **PerCP** | **Biolegend** | **GK1.5** |
| **CD3** | **BV421** | **Biolegend** | **KT3.1.1** |
| **IL4** | **VioB515** | **Miltenyi** | **Cat# 130-108-167** |

**Supplementary Table 1 – Flow cytometry antibodies.**

**
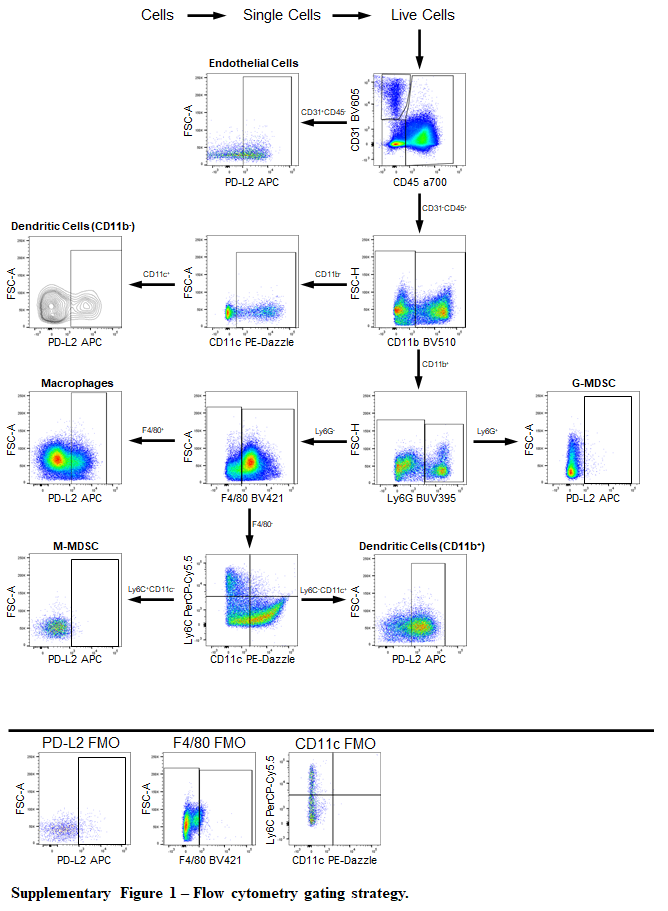
**

**Supplementary Figure 1 – Flow cytometry gating strategy.**

**
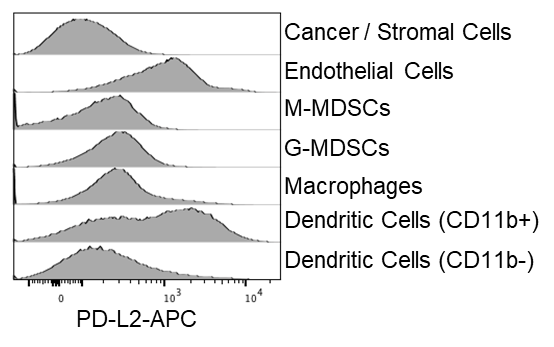
**

**Supplementary Figure 2 – PD-L2 expression on multiple cell types within the pancreatic TME.** Representative histogram of flow cytometry analysis of PD-L2 expression on different cell types within Panc47 FAK-wt tumours.


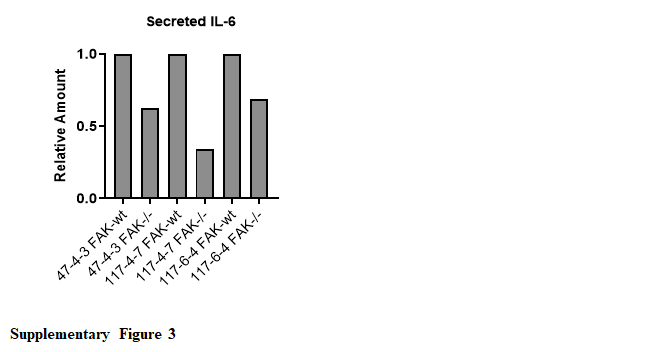


**Supplementary Figure 3 – Multiple FAK-depleted CRISPR clones derived from murine PDAC cell lines exhibit FAK-dependent regulation of IL6.** Forward-phase anti-IL6 protein array analysis of media conditioned by FAK-wt and FAK-/- PDAC cell line pairs.

**
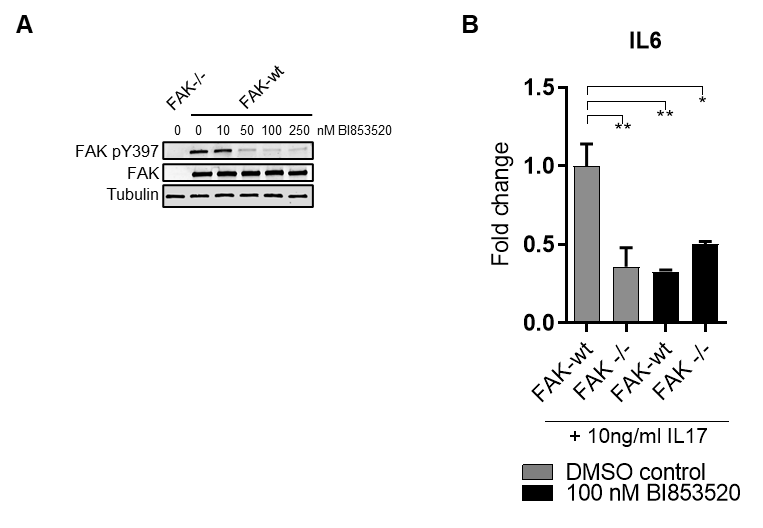
**

**Supplementary Figure 4 – IL6 expression requires FAK kinase activity.** (A) Representative western blot of whole cell lysates isolated from Panc47 FAK-wt cells +/- treatment with increasing concentrations of the FAK kinase inhibitor BI 853520. Lysates isolated from Panc47 FAK-/- cells used as a control. Membrane probed with anti-FAK pY397, anti-FAK, and anti-tubulin antibodies. (B) Anti-IL6 ELISA using media conditioned by Panc47 FAK-wt or FAK-/- cells simulated with IL17 and treated with either DMSO or 100 nM BI 853520.


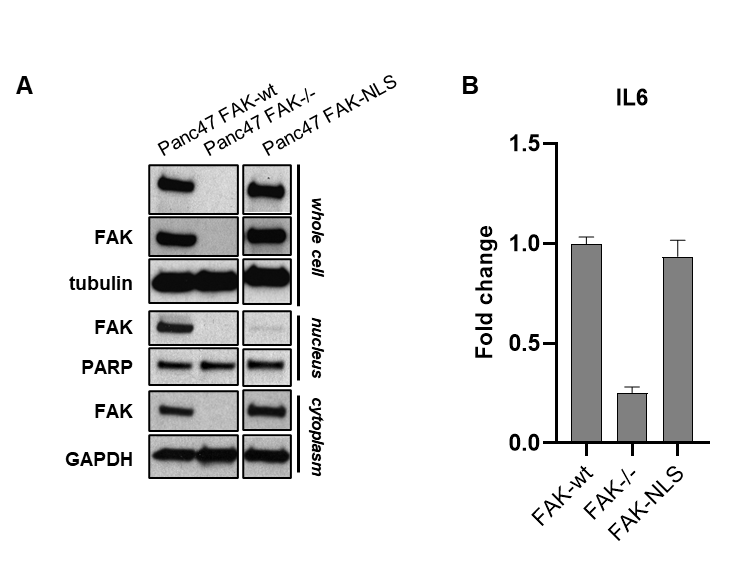


**Supplementary Figure 5 – Secretion of IL6 is not dependent on FAK nuclear translocation.** (A) Western blot of whole cell lysates, nuclear extracts and cytoplasmic extracts isolated from Panc47 FAK-wt, FAK-/- and FAK-NLS cells. All samples were run on the same gel, but samples not relevant to the study have been removed as indicated by a break in the image. (B) Anti-IL6 ELISA using media conditioned by Panc47 FAK-wt, FAK-/- and FAK-NLS cells.


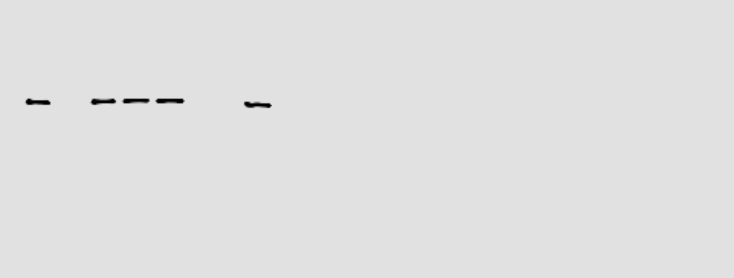

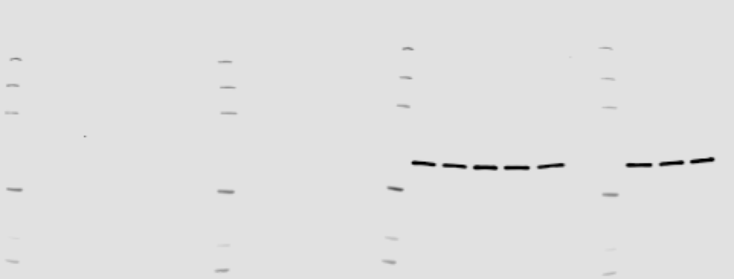


FAK

Tubulin

FAK-wt

FAK-/-

FAK-wt CTL shRNA

FAK-wt IL6 shRNA1

FAK-wt IL6 shRNA2


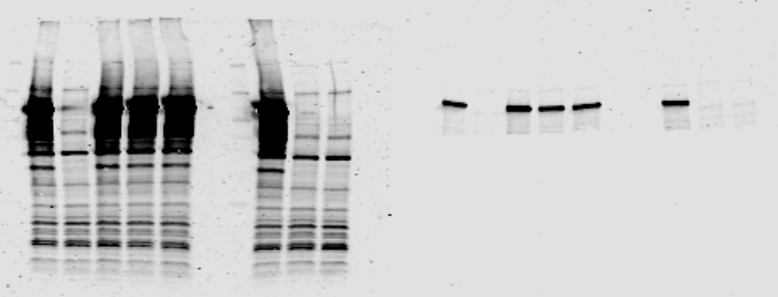


FAK pY397

**Supplementary Figure 6 – IL6-depletion does not affect FAK expression.** (A) Representative western blot of whole cell lysates isolated from Panc47 FAK-wt, FAK-/-, FAK-wt CTL shRNA, FAK-wt IL6 shRNA1, and FAK-wt shRNA2 cells. Membrane probed with anti-FAK pY397, anti-FAK, and anti-tubulin antibodies.
